# Virtual Reality Exercise as a Coping Strategy for Health and Wellness Promotion in Older Adults during the COVID-19 Pandemic

**DOI:** 10.3390/jcm9061986

**Published:** 2020-06-25

**Authors:** Zan Gao, Jung Eun Lee, Daniel J. McDonough, Callie Albers

**Affiliations:** 1 School of Kinesiology, University of Minnesota-Twin Cities, Minneapolis, MN 55455, USA; mcdo0785@umn.edu (D.J.M.); alber505@umn.edu (C.A.); 2 Department of Applied Human Sciences, University of Minnesota, Duluth, MN 55812, USA; junelee@d.umn.edu

**Keywords:** cognition, fall prevention, motor ability, obesity, psychological outcomes

## Abstract

The December 2019 COVID-19 outbreak in China has led to worldwide quarantine, as recommended by local governments and the World Health Organization. Particularly affected are older adults (i.e., those aged ≥ 65 years) who are at elevated risk for various adverse health outcomes, including declines in motor ability and physical activity (PA) participation, increased obesity, impaired cognition, and various psychological disorders. Thus, given the secular increases in the older adult population, novel and effective intervention strategies are necessary to improve physical activity behaviors and health in this population. Virtual reality (VR)-integrated exercise is a promising intervention strategy, which has been utilized in healthcare fields like stroke rehabilitation and psychotherapy. Therefore, the purpose of this editorial is to synthesize recent research examining the efficacy and effectiveness of VR exercise in the promotion of favorable health outcomes among the older adults. Results indicate the application of VR exercise to facilitate improved physical outcomes (e.g., enhanced motor ability, reduced obesity), cognition and psychological outcomes. VR exercise has also been observed to be an effective intervention strategy for fall prevention in this population. Future research should employ more rigorous research designs to allow for a more robust quantitative synthesis of the effect of VR exercise on the preceding outcomes to elucidate which type(s) of VR-based PA interventions are most effective in promoting improved health outcomes among older adults. Findings from this study will better inform the development of technology-savvy PA programs for wellness promotion in older adults who practice social distancing and exercise from home under the unprecedented global health crisis.

## 1. Introduction

The December 2019 novel coronavirus (COVID-19) outbreak in China has infected more than 9.97 million people and resulted in over 480,000 deaths worldwide [1], which has led to global quarantine as recommended by local governments and the World Health Organization. Indeed, quarantine can help mitigate individuals’ exposure to COVID-19 and, therefore, minimize the risk of contracting the virus. However, the quarantine orders have created many national challenges that have had profound impacts on financial, physical, psychological, and emotional health among people of all ages [2,3]. Particularly affected are older adults (i.e., those 65 years and older) who are more likely to suffer from serious COVID-19 illness. In fact, 8 out of 10 deaths reported in the U.S. have been in older adults [4]. This may be partially attributed to compromised immune systems with age, making it harder to fight off coronavirus diseases and infection. In the past 30 years, researchers have found regular physical activity (PA) participation to have beneficial effects on older adults’ health and wellbeing [5,6].

From 2020 to 2030, the number of older adults in the U.S. is expected to increase from 56 million to 74 million, which amounts to one in five Americans [7]. This generation has higher rates of chronic disease and disability compared to any other generation [8], and studies have shown that the four most common poor health conditions seen in older adults are decreased motor ability, increased obesity, impaired cognition, and psychological disorders, which lead to a lower quality of life [9,10]. For example, an inactive lifestyle, along with a natural decline in physiological markers with age, contributes to a loss in muscle strength and balance [9], and, through deterioration of motor abilities, older adults’ risk for falls and fractures increases [8]. Furthermore, the prevalence of obesity in older adults puts them not only at higher risk for developing cardiovascular diseases but also acquiring a disability and remaining physically impaired [10]. Cognitive impairment is a health concern that makes it difficult for older adults to live independently and also places them at a higher risk for falls [9]. For instance, it has been shown that older adults with cognitive impairment are twice as likely to have a fall compared to older adults without impaired cognition [9]. Lastly, depression, anxiety disorder, and dementia are the most prevalent psychological problems in older adults [10]. It is dismaying that 21% of older adults report experiencing symptoms of anxiety that contribute to significant distress, lower quality of life, and a higher chance of having depression [11]. Thus, it is important to develop and implement effective intervention strategies that can prevent or reverse these adverse health outcomes in order to improve older adults’ quality of life.

Given that many are experiencing stressful life challenges under the COVID-19 pandemic crisis, it is imperative to develop innovative and effective PA intervention programs that reduce stress and promote health and wellbeing in older adults [12,13]. One innovative intervention strategy that has shown promise in the healthcare field is virtual reality (VR)-based PA interventions [14]. However, reviews investigating the effectiveness of VR in the promotion of better health outcomes in older adults are scarce [15]. Therefore, the purpose of this editorial was to determine the efficacy and effectiveness of VR exercise in aiding healthy older adults to have increased motor ability, reduced obesity, improved cognition, and better psychological outcomes. As known, VR is a new and engaging technology that has received limited research with regards to health promotion in older adults. The findings of this paper may provide healthcare practitioners and researchers with valuable information on the utility of VR that they could apply in community and home settings under challenging circumstances.

## 2. Fundamentals of VR

One intervention strategy which has shown promise for promoting healthy aging among older adults is VR-integrated exercise [16,17]. VR exercise is a novel and innovative technology, which immerses individuals in a computer-generated, multi-sensory, three-dimensional world wherein they interact with the virtual environment using either a headset and/or exercise equipment [18,19,20]. VR technology can be dichotomized by immersion (i.e., immersive and non-immersive). Immersive VR typically requires the use of a head-mounted display (e.g., Oculus Rift, Menlo Park, CA, USA) or an entire room display which encloses the user (e.g., cave automatic virtual environment (CAVE)) [21]. Non-immersive VR, on the other hand, offers users a computer-generated world which typically uses a desktop or projector [18]. Examples of non-immersive VR include the Nintendo Wii Switch and the Xbox 360 Kinect, which are often more cost-effective and better for use in the home setting compared to immersive VR equipment [22,23,24,25,26,27,28].

VR technology is currently used in a variety of health field areas, such as psychotherapy and stroke rehabilitation [14], and has been shown to be effective in improving balance and overall health and promoting weight loss in older adults [9,14,16,29]. For instance, VR has been implemented within therapeutic programs for phobias related to height and public speaking, in which patients were immersed into an environment where they progressively worked on their fears [30,31]. Furthermore, VR exercise has been successfully used within rehabilitation settings for motor learning following a stroke, which led to patients’ increased brain plasticity [17,32]. VR has also been shown to be effective in exercise promotion, which led to multiple health benefits, including reduced obesity and anxiety, as well as improved cognition [9,16,29]. Additionally, studies have suggested that VR consisting of cognitive behavioral treatment could aid in weight loss and alleviation of psychological disorders [11,33]. Along with all these health benefits, VR also presents itself as a potential candidate for promoting leisure activity. Participants who were immersed into nature via VR while using a traditional exercise bike reported that it was much more enjoyable than traditional exercise biking alone [34]. The application of VR has been shown to have positive benefits on older adults’ physical and mental health; however, these findings are still limited. Therefore, more innovative and technology-savvy interventions need to be employed to help control obesity rates and poor health concerns in this population.

## 3. Effects of VR on Physical Outcomes in Older Adults

### 3.1. VR and Motor Ability in Older Adults

Due to aging, older adults naturally exhibit decreased motor ability, including compromised coordination, balance, muscular strength, and speed [22]. In general, VR exercise has demonstrated positive effects on the preceding components of older adults’ motor ability by engaging older adults’ motor skills and promoting sensorimotor learning and cortical plasticity to improve their motor ability. For example, a home-based VR intervention, which used an Xbox 360 gaming console and Your Shape Fitness Evolved software and consisted of Tai Chi and Yoga exercise programs, indicated positive effects of VR exercise on older adults’ motor ability outcomes, such as hip muscle strength and balance control [22]. Furthermore, significantly improved muscle strength as assessed by hand grip dynamometry and an arm curl test and improved balance measured by a postural sway test were evident in another study that implemented a three-dimensional VR kayak program [9]. While these two studies had muscular strength as an outcome measure which significantly improved, one looked at hip strength and the other used grip strength. Due to this difference, the effect of VR on targeted muscle strength is inconclusive, and more research is needed in the future.

Rehabilitation methods (e.g., therapeutic exercise) have been employed extensively with the aim of improving older adults’ motor ability. However, current rehabilitation methods with this aim often fail to account for the characteristics and needs of patients and, consequently, the patients often do not see rehabilitative success in the real world [35]. Findings suggest that the learning of new skills and activation of brain plasticity are enriched when a patient is placed in an appropriate environment that resembles real life [35]. For example, in a recent study that employed an immersive VR intervention (the CAVE), the scenario placed participants in an apple orchard, where they had to reach out as quickly as possible to grab the virtual apple then place it in the basket to score points [35]. The results demonstrated a gradual increase in scores and improved postural stability, which is an important component of motor ability. Overall, existing VR exercise programs were all shown to significantly improve older adults’ motor ability through increased balance. With improved balance control, older adults can achieve better health outcomes, such as reduced falls. However, research examining the effect of VR exercise on strengthening the larger musculature (e.g., hips, arms) is needed to determine if it is an effective intervention strategy for improving motor ability in older adults.

### 3.2. VR and Obesity in Older Adults

Studies show that over one third of older adults are obese, and the prevalence is steadily increasing [36]. This calls for effective and innovative intervention strategies to manage and prevent obesity in older adults. While VR exercise’s utility for weight loss and control is relatively new, it is well established that technology-based interventions targeting weight loss are scalable and cost-effective [37]. For example, Manzoni and colleagues [33] and Thomas and Bond [37] examined the efficacy of VR-integrated cognitive-behavioral treatment (CBT) for reducing obesity among older adults. CBT is a type of psychotherapy commonly used to help treat eating disorders, which aims to change individuals’ thinking patterns using a goal-oriented approach [37], whereas VR-integrated CBT aims to teach problem-solving techniques and reduce body weight and problematic eating. Manzoni and colleagues [33] utilized the NeuroVR open space software to station participants in real-world environments where they had to handle situations of daily living, such as working out at a gym, shopping at the grocery store, or dining at a restaurant. The researchers observed at one-year follow up that the VR group displayed consistent weight loss maintenance, whereas the control group gained back most of their lost weight. Additionally, Thomas and Bond [25] conducted research using a VR-based behavioral weight loss program (Second Life Virtual World), in which participants learned to navigate difficult situations. Although the sample size in this study was small, the results suggest that VR may be more beneficial for long-term weight loss compared to traditional, face-to-face treatments.

Beyond VR-integrated CBT’s implications for weight loss, VR may also encourage weight loss indirectly through the promotion of PA. Wii Fit, for example, is readily accessible, affordable, and motivating for older adults and has shown promise for promoting PA and weight loss in this population. For example, one study observed Wii Fit Sports to increase daily energy expenditure and time spent in moderate to vigorous PA in older adults at risk for obesity [38]. Although no significant correlations could be made due to the small sample size, the findings showed modest weight loss and enjoyment among participants while they engaged in the exercise, which may be promising for long-term adherence [38]. While VR-integrated CBT studies [33,37] have reported chronic effects on weight loss as compared to controls, the preceding Wii Fit study [38] primarily targeted participants’ attitudes toward PA, which indicated VR exercise to be more a more engaging form of exercise compared to traditional exercise. 

Overall, findings suggest VR-integrated CBT is effective for assisting older adults in weight loss maintenance for months after the cessation of the intervention programs [33,37]. Further, Wii Fit Sports Games increased participants’ PA levels and PA-related enjoyment following an 8-week program. Notably, however, given the small sample size and short intervention length, these findings warrant further empirical support [38]. VR-based exercise interventions like CBT and Wii Fit exercise programs are highly accessible, cost-effective, and motivating strategies, which show promise for obesity reduction in older adults. However, further research addressing the preceding research gaps are needed.

## 4. Effects of VR on Cognition in Older Adults

Declines in cognitive ability is a part of normal aging and may eventually develop into cognitive disorders [11]. VR has shown promise for improving cognitive functions, such as executive function, visuospatial processing, and memory [9]. Specifically, VR interventions like immersive memory training and a three-dimensional kayaking exercise program significantly improved older adults’ short- and long-term memory [9,39]. Further, another study observed a 6-week VR kayaking program to significantly improve cognitive older adults’ cognitive functioning, including executive functions, conceptual thinking, concentration, attention, visuoconstructive skills, working memory, mathematical calculations, language, and orientation [9]. Results indicated these cognitive domains to significantly improve from pre- to post-intervention only in the VR experimental group. Another study indicated that VR exercise may also be a promising tool for improving cognitive functioning using VR memory training [39]. In this study, participants in the VR group used a head-mounted display and a joystick to maneuver along city paths within the immersive VR environment and were then asked to memorize and recall those paths. Findings from the neuropsychological tests showed significant improvements in overall cognitive functioning and verbal memory. Notably, only small, non-significant improvements in executive functioning and visuospatial processing were observed. This may be attributable to some of the tests requiring drawing pictures, and not all participants may have had the natural drawing abilities needed to adequately perform on these tests.

Although these two studies [9,39] targeted similar cognitive domains, such as executive function, memory, and visuoconstruction/visuospatial skills, as health outcomes, the two differed in terms of the level of improvement in such outcomes. Possible explanations for these differences include different samples and intervention components and inconsistency in the employed cognitive domain tests. Therefore, more research with consistent intervention components and testing is warranted to determine if VR exercise truly facilitates significant improvements in these cognitive functions. However, memory was observed to significantly improve in both studies. In sum, VR exercise shows promise for improving cognitive functioning and memory in older adults as well as other cognitive outcomes, but further research is warranted to confirm this. With an increase in cognitive function and ability, older adults will experience improved mental health outcomes and exhibit a lower risk for falls. 

## 5. Effects of VR on Psychological Outcomes in Older Adults

Findings indicate that over 21% of older adults experience anxiety symptoms [11]. The use of VR exercise has shown promise for decreasing anxiety and depression in older adults, which may translate to improved overall mental health outcomes in this population [29]. This preliminary review identifies and examines five eligible studies, which reported that VR exercise programs can relieve feelings of anxiety and depression and increase enjoyment and daily energy levels [29]. For example, one study had 54 older female participants undergo either a group-based exercise program or a VR-based Tai Chi exercise program. The investigators observed the VR exercise group to report significantly greater decreases in anxiety and depression compared to the traditional exercise program. On the other hand, one study not included in the review utilized the geriatric depression scale and observed no significant differences in these outcomes following a VR-based, Wii Fit Balance intervention [40]. There is also the possibility of using VR with CBT to decrease anxiety in older adults. In another preliminary review, which examined three meta-analyses to determine the potential of VR-enhanced CBT in treating anxiety disorders in older adults [11], the authors revealed that the number of CBT randomized controlled trials in older adults was half that of studies on younger adults and none have been designed to explore VR-enhanced CBT for adults 65 and older. Since VR-enhanced CBT has been successful in treating anxiety disorders in younger adults, Grenier et al. [11] proposed a pilot study that investigates the efficacy of an 8-week CBT program which integrates VR. The treatment will teach participants how to cope with the triggers and episodes of anxiety.

In sum, VR has been purported as a promising tool for facilitating better mental health outcomes in older adults when combined with CBT and for its ability to relieve feelings of anxiety and depression. However, more supporting empirical evidence is needed in this field of inquiry, considering that only one empirical study and two preliminary reviews were identified. Both the Wii Fit and VR-based Tai Chi studies used anxiety and depression as the mental health outcomes. However, compared to controls, only the VR-based Tai Chi PA program prompted significant improvements in feelings of anxiety and depression. Conversely, the Wii Fit program, compared to the control group, observed some improvement in feelings of anxiety and depression, though statistical significance was not reached for either outcome. Differences in outcomes between studies may be explained by differences in modality, duration, intensity, and/or frequency of the exercise programs. Thus, more research is needed to discern the effectiveness of VR exercise in the promotion of improved psychological outcomes in older adults, such as depression and anxiety.

## 6. Effects of VR on Rehabilitative Outcomes in Older Adults

Approximately 30% of older adults experience at least one fall each year, and those that have a fallen are at increased risk of falling again [41]. Older adults who have a history of falls tend to have significantly lower muscle strength in their hip musculature [22]. PA has been shown to improve muscular strength and balance, and, therefore, reduce the risk of falls among the elderly [42]. Research has identified two main types of VR-based exercises that are related to older adults’ reduced fall rates: VR-based treadmill exercises and Wii Fit exercises. To date, two studies have examined the effects of VR-based treadmill exercise, both of which found significant decreases in the incidence of falls in the VR training group compared with a traditional treadmill exercise group [43,44]. With regard to Wii Fit exercise, studies suggested that both immersive and non-immersive Wii Fit exercise can decrease older adults’ risk for falls by improving their motor functioning, such as by improving their center of balance [14,45]. Chiarovano et al. [45] used immersive VR (Oculus Rift DK2 VR headset) in conjunction with the Wii Fit Balance Board and the BalanceRite application, while other researchers [14] used a non-immersive Nintendo Wii Fit exercise wherein participants played Ski Slalom, Table Tile, and Balance Bubble. Findings suggested that, through having older adults perform the dual task of working on postural stability as well as respond to powerful visual stimuli, older adults increased their capacity for attention demands and decreased their risk of falls. These findings support the effectiveness of VR exercise interventions in reducing fall rates and improving balance in older adults. Thus, the use of VR exercise training can be a more effective fall prevention tool compared to treadmill exercise training alone through increased balance and speed and the teaching of reactive strategies.

It has been reported that age-related cognitive declines increase older adults’ risk for falls, which are a major contributor to morbidity and mortality rates in this population [43]. For instance, 60–80% of older adults who have cognitive impairments report at least one a year. These falls often occur due to compromised executive functioning and, therefore, navigation, causing them to trip over obstacles and basic objects [43]. Therefore, improving cognition is of paramount importance for reducing the risk of falls and improving quality of life in older adults. One study [43] that examined the use of VR treadmill exercise as an intervention strategy to reduce falls also targeted cognitive functioning. In detail, the VR simulation was composed of real-life situations and challenges, such as obstacles and distractions, in order to enhance older adults’ cognitive functioning (i.e., executive function and attention) while walking. Executive functioning and attention play a major role in obstacle clearance and are, therefore, essential in the prevention of falls. The findings from this study indicate that treadmill training concurrent with VR exercise is more effective than treadmill exercise alone for improving cognitive functioning and, therefore, reducing falls among older adults. In addition, findings from two other studies [22,45] showed that both immersive and non-immersive VR treadmill exercise and the Wii Balance Board were effective for reducing rates of falls in older adults by lessening the severity of falls and teaching more effective fall prevention strategies.

Fear of falling in older adults entails an intense fear of standing or walking. The prevalence of this phenomenon is 24–55% in older adults and up to 92% in older adults who have experienced at least one fall [46]. Serious consequences come with fear of falling, including decreased social interactions, physical injury, reduced quality of life, and accidental death, which further supports the need for effective exercise-based therapeutic interventions. Current available treatments include traditional exercise interventions and protectors worn at the hip. However, these methods have only shown minimal effects and do not consider the psychological aspect of the fear of falling [46]. That said, VR exercise has shown promise for addressing the fear of falling in older adults. For instance, Levy and colleagues [46] examined the effect of immersive VR games in participants who reported having a fear of falling, such as fighting off enemies by moving their hands and washing a window with foam. A questionnaire regarding the activities of daily life (e.g., getting out of bed, putting on clothes) demonstrated significant improvements in older adults’ fear of falling after the VR exercise intervention compared to a control group. These findings showed promise for the utility of VR-based exercise interventions for successfully reducing older adults’ fear of falling and, thus, improving their motor ability and overall quality of life. Noteworthy is the fact that this study had a small sample size and, therefore, more research is needed to further support these findings.

## 7. Practical Implications

VR is a promising tool for effective treatment in the rehabilitation setting. By implementing non-immersive VR on the treadmill or immersing a patient into a realistic environment, such as a city or park setting with a head-mounted display or within the CAVE, physical and occupational therapy sessions can be enhanced, subsequently increasing the chance of successful adaptation to the real world [35]. Participants also found that exercising on a stationary bike with VR that immersed them into nature was significantly more enjoyable than traditional biking without VR [34,47]. Since VR was found to be an engaging activity for older adults, this could lead to better adherence to a rehabilitation program, which in turn may lead to better health outcomes in patients.

VR exercise interventions also include home-based interventions, such as VR-based Tai Chi and yoga programs [22]. The use of at-home rehabilitation techniques would lead to more effective rehabilitation, as older adults can receive real-time feedback from home by using VR during times in which they are not at the clinic. This may be especially important during the COVID-19 pandemic, as older adults may wish to remain quarantined in their homes given their increased risk of contracting the virus. Home-based VR exercise interventions can also help relieve stress from healthcare services with the surge of baby boomers reaching older age. This reduction in overscheduling for physical and occupational therapists may allow them to provide better care during their sessions. Further, during in-person appointments, VR exercise can be supplemented to increase patients’ exercise motivation and enjoyment.

## 8. Directions for Future Research

Though some studies support VR exercise’s effectiveness in promoting better health outcomes among older adults, they are not without limitations. For example, older adults’ success in using VR-integrated exercise may be limited by perceptual, mental, and physical declines that naturally come with age [10]. Thus, these individuals may be discouraged from participating in VR exercise interventions and may negatively impact retention rates in such studies. Second, many of the included studies had small sample sizes (≤30 participants), which may have affected the external validity of the findings. Additionally, the implemented VR exercise interventions varied greatly, in that immersive and non-immersive VR-integrated exercise equipment and VR-enhanced CBT, among other intervention strategies, were used across studies. This renders it difficult to confidently conclude that all VR exercise modalities and programs can facilitate better health outcomes in older adults. As such, we recommend more research be conducted in this area of inquiry to better discern which VR interventions are the most effective among older adults.

Future studies should address the research design issue observed in most studies by increasing sample sizes [48]. More research focusing on the mental health problems seen in older adults is also needed. In addition, there is a need for more research investigating the effectiveness of VR exercise programs on older adults’ weight loss, as VR exercise has only recently been applied as a means for weight control. In addition, examining the motivation to maintain or increase PA participation [49] during leisure time among older adults using VR at homes or community centers is warranted. Finally, as stated above, health professionals need to determine which specific types of VR are most effective for improving health outcomes in healthy older adults. This may include determining factors, such as modality, intensity, duration, and frequency, as well as VR exercise setting(s) most suitable for older adults.

## 9. Summary

The purpose of this paper was to explore the potential of using VR exercise as a coping strategy for health and wellness promotion in older adults during the COVID-19 pandemic. VR is an emerging technology that is a valuable tool for healthy aging in older adults. Empirical studies support that VR leads to improvements, although not always significant, in the four most common health concerns seen in older adults: decreased motor ability, increased obesity, impaired cognition, and various psychological disorders. Across studies, findings demonstrate that VR exercise interventions lead to significant improvements in older adults’ balance and memory, which contribute to a lower risk for falls. Given the secular increases in the older adult population, healthcare services must be equipped to meet their specific health needs. Indeed, chronic disease and disability prevalence in this generation of older adults can be compared to any other generation and VR is purported to be a valuable intervention tool and strategy in rehabilitation and/or home settings in this population. Integrating VR into physical and occupational therapy may serve to minimize stress in clinicians and patients by allowing patients to engage in VR-based rehabilitation from home. Further, compared to traditional exercise intervention strategies, VR exercise has been shown to be more effective in leading to more significant and faster recoveries. This may be partially attributed to VR’s engaging nature, making it well tolerated by older adults. Additionally, VR exercise interventions may have multiple health benefits pertaining to older adults’ motor ability, obesity status, cognition, and psychological outcomes. However, much more research is needed to investigate this novel treatment strategy among older adults. It is especially imperative for health professionals to deliver exercise programs remotely due to social distancing under COVID-19 and for possible future pandemic crises.
